# Efficiency of probiotics in elderly patients undergoing orthopedic surgery for postoperative cognitive dysfunction: a study protocol for a multicenter, randomized controlled trial

**DOI:** 10.1186/s13063-023-07167-6

**Published:** 2023-02-25

**Authors:** Xiaoyi Zhang, Yuwen Chen, Ying Tang, Yizhe Zhang, Xiao Zhang, Diansan Su

**Affiliations:** Department of Anaesthesiology, Renji Hospital, Shanghai Jiaotong University School of Medicine, 160 Pujian Road, Shanghai, 200127 China

**Keywords:** Probiotics, Orthopedic surgery, Postoperative cognitive dysfunction, Randomized controlled trial

## Abstract

**Background:**

Postoperative cognitive dysfunction (POCD) refers to a neurological dysfunction after a major surgery and anesthesia. It is common in elderly patients and is characterized by impairment in consciousness, orientation, thinking, memory, and executive function after surgical anesthesia. However, at present, there is no definite preventive or treatable strategy for it. Previous animal experiments showed that giving probiotics to mice before operation can prevent POCD, but there is a lack of clinical evidence. This study aims to intervene with the intestinal flora imbalance using probiotics during the perioperative period to reduce the incidence of POCD in elderly patients after orthopedic surgery and to provide new ideas and methods for the clinical prevention and treatment of POCD.

**Methods:**

A multicenter, double-blind, placebo-controlled clinical trial will be performed to evaluate the efficacy of probiotics in elderly patients undergoing orthopedic surgery. Participants (*n* = 220) will receive probiotics (Peifeikang, Live Combined Bifidobacterium, 210 mg per capsule, twice a day, four capsules each time, which contains Bifidobacterium longum, Lactobacillus acidophilus and Streptococcus faecalis no less than 1.0 × 10^7^ CFU viable bacteria respectively) or placebo from 1 day before surgery to 6 days after surgery. Neuropsychological tests will be performed 1 day before surgery and 1 week and 1 month after surgery. The main outcome of this study is the incidence of POCD 7 days after surgery. Our secondary objective is to assess the incidence of POCD 1 month after surgery; the cognitive status will be determined based on a telephone interview and will be evaluated via TICS-m; postoperative delirium will be assessed 7 days after surgery using the Confusion Assessment Method (CAM).

**Discussion:**

Discovering the correlation between the intestinal microbiota and POCD is an important breakthrough. Based on the key role of the intestinal microbiota in other cognitive disorders, we hope that probiotics can reduce its incidence in elderly orthopedic patients.

**Trial registration:**

ClinicalTrials.gov NCT04017403. Registered on August 15, 2019.

**Supplementary Information:**

The online version contains supplementary material available at 10.1186/s13063-023-07167-6.

## Introduction

### Background and rationale

Postoperative cognitive dysfunction (POCD) refers to cognitive function changes, such as decreased learning and memory ability and inability to concentrate, after anesthesia and surgery. In severe cases, personality changes and decreased social behavior ability are observed [1]. Some patients even develop irreversible cognitive impairment [2]. POCD increases the hospitalization time of patients, prolongs their stay in intensive care units, increases their medical expenses, and increases the mortality of patients 1 year postoperatively [3]. POCD have become a widespread concern of clinicians and patients. Clinical studies showed that its incidence after a major surgery is relatively high, and major surgeries are often accompanied by blood redistribution caused by factors, such as bleeding and hypotension, resulting in intestinal mucosal ischemia [4, 5]. Intestinal mucosal ischemia is an important cause of intestinal flora imbalance [6, 7]. Patients with severe diseases (including sepsis, acute respiratory distress syndrome, and multiorgan failure) and those undergoing intestinal surgery are prone to changes in the intestinal flora. They are more likely to have intestinal flora disorders due to long-term applications of antibiotics, unstable hemodynamics, long-term parenteral nutrition, and other factors [8]. Such patients are more prone to POCD [9]. Its incidence in patients undergoing intestinal surgery is also relatively high. Tan et al. [10]. found that its incidence after an endoscopic or open colectomy is approximately 47%.

Studies in our laboratory proved that [11] giving probiotics to mice before operation can prevent POCD. However, at present, there is a lack of clinical evidence. In this study, probiotics will be used to intervene with the intestinal flora imbalance perioperatively in order to reduce the incidence of POCD and provide new ideas and methods for its clinical prevention and treatment.

## Methods

### Study design and setting

In this study, we will recruit 220 participants from Renji Hospital, School of Medicine, Shanghai Jiaotong University, Shanghai Guanghua Integrated Traditional Chinese and Western Medicine Hospital, and Shanxi Jincheng Hospital. The clinical trial was approved by the Ethics Committee of Renji Hospital (2018–232) and registered at ClinicalTrials.gov on August 15, 2019 (No. NCT04017403). The trial was conducted according to the recommendations of the Standard Protocol Items: Recommendations for Interventional Trials (SPIRIT) (supplemental file, SPIRIT checklist). We presented the trial registration data in the form of supplemental data.

This is a multicenter, double-blind, randomized, placebo-controlled, interventional superiority clinical study. We will observe the effect of perioperative probiotics on the postoperative cognitive function of elderly patients undergoing orthopedic surgery.

### Objectives

The purpose of this study is to determine whether the perioperative intervention with probiotics on intestinal flora can reduce the occurrence of POCD and to provide new ideas for its clinical prevention and treatment. Our primary objective is to assess the incidence of POCD 7 days after surgery. Our secondary objective is to assess the incidence of POCD and postoperative delirium (POD) 1 month and 7 days after surgery, respectively. We predict that probiotic intervention will reduce its incidence in elderly patients undergoing orthopedic surgery.

**Fig. 1:**
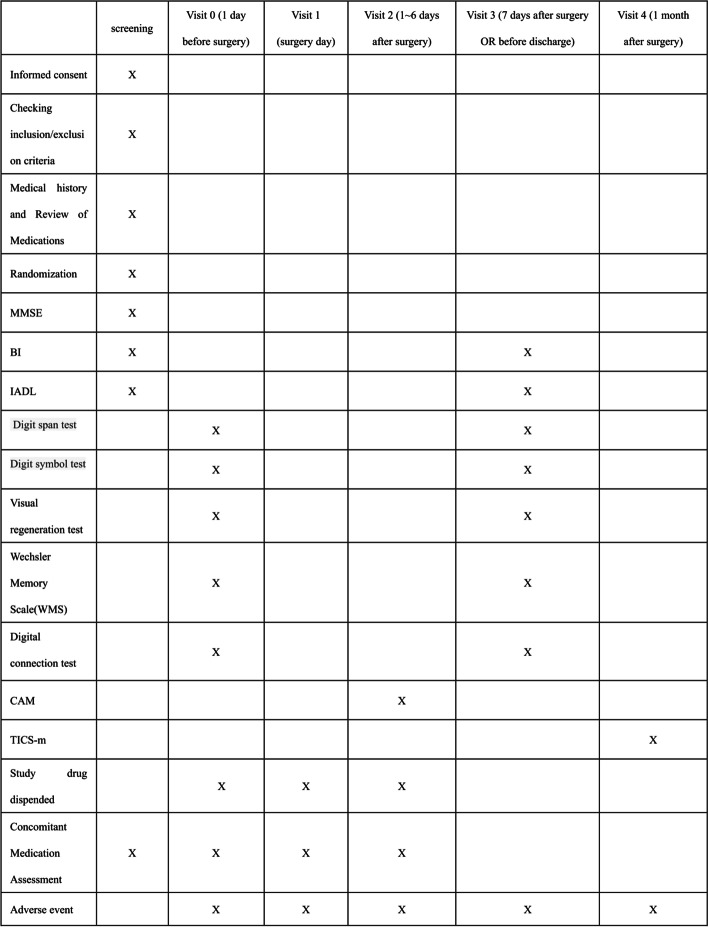
The schedule of major study events that follows the Standard Protocol Items: Recommendations for Interventional Trials (SPIRIT) figure of enrolment, interventions, and assessments

The schedule of the major study events for each visit is shown in Fig. 1. Patients who are scheduled to undergo orthopedic surgery will be included in this study. The entire process of recruitment and consenting of study participants by members of the research team will be consistent with good clinical practice (GCP) recommendations. Patients will be recruited mainly from the three centers.

### Eligibility criteria

The inclusion/exclusion criteria are summarized in Table 1.

**Table 1:** Inclusion/exclusion criteria

Inclusion criteria	Exclusion criteria
1) Age is greater than or equal to 65 years old 2) Can communicate normally 3) Elective knee ankle, hip replacement, or lumbar open reduction and internal fixation under general anesthesia 4) ASA graded at I–II level 5) Patient or family informed consent	1) Have a brain disease, or have a history of brain disease 2) MMSE check of less than 24 points 3) History of neurological and psychological disorders including AD, stroke, psychosis 4) Serious hearing or visual impairment 5) Preoperative systolic blood pressure > 190 mmHg, or diastolic blood pressure > 100 mmHg 6) The patient or family refuses

### Consent

After assessing the patient’s eligibility for inclusion, his/her informed consent will be obtained. A designated doctor will explain the experiment in detail to interested participants and provide them with informed consent. Participants will have at least 24 h to decide whether they want to participate in the trial. An informed consent form will be signed by the participants or their trustee or guardian; it can be withdrawn at any time during the trial. A written informed consent and patient baseline data will be obtained before randomization. Moreover, participants will be encouraged to contact the research team if they have any health concerns during the trial.

We will collect baseline data from patients and randomize allocation using Statistical Analysis Set (SAS) software. Furthermore, participants can contact our team if they have any health concerns during the trial. The recruitment and consenting of study participants by the research team are in line with the GCP guidelines. During the clinical trial, the researchers will immediately report any serious adverse events, whether related to the probiotics under study or not, to the director in charge of the clinical trial of the research institution and contact Professor Diansan Su or Dr. Xiaoyi Zhang.

No ancillary study is planned, and we have no plans to collect additional participant data or biological specimens outside of what is mentioned in this protocol.

Participant information will be confidential and managed according to the Data Protection Act, NHS Caldecott Principles, The Research Governance Framework for Health and Social Care, and the conditions of Research Ethics Committee approval.

### Randomization and blinding

#### Randomization

Randomization will occur during or immediately after the baseline visit. An independent statistician from a third-party statistical agency will use SAS statistical software (SAS Institute, Inc.; Cary, NC, USA) to generate random sequences, perform random grouping, and code drugs; patients will be randomly categorized into two groups in a 1:1 ratio per the randomized sequence table. The participants who received placebo will be considered as controls. The distribution scheme of hiding and implementing known as the opaque envelope method will be used to hide random sequences. The random code of the drug will be the unique identification code of the participants. The letter will be collected together with the case report form (CRF) after the end of the trial.

#### Blinding

Blindness will be ensured between the investigators (doctors) of the study and subjects (patients). In the drug preparation stage, the placebo is specially made by the probiotic manufacturer. Except that it does not contain probiotics, other excipients are the same, and the appearance and packaging of the drugs will be consistent. For blinding purposes, participants of the two groups will receive the same number of capsules daily and will be unaware of their own and others’ grouping before, during, and after the orthopedic surgery. As the medication will be encapsulated and provided by an independent nurse per the random codes, the investigator, intraoperative attending anesthesiologist, evaluator, and data analyst will be unaware of the allocation until the study is completed or a patient is intentionally unblinded. The aforementioned independent statistician will not be involved in the study process and statistical analysis of the test results.

An unblinding (emergency) envelope contains the information of the drugs to be taken by the subjects with their corresponding numbers. This will be sent to the researchers together with the study drug for use in case of emergency. It is forbidden for clinical efficacy evaluators to ask drug managers and subjects for product information to avoid influencing the efficacy evaluation due to subjective factors. In case of a medical emergency, which requires the identification of an individual patient’s treatment, the investigators will be permitted to open the respective emergency envelope. A justification must be documented in the patient’s medical record and CRF. Serious adverse events will recorded for further analysis at the end of the trial, and investigators will explore the correlation between these events and patients’ healthcare records.

### Interventions

Figure 1 shows the schedule of major study events for each visit. Elderly patients planning to undergo orthopedic surgery will be included in this study. The surgery will be performed within 2 days after screening. The drug will be used a day before operation until 6 days postoperatively. The probiotic group will be treated with Peifeikang (Bifidobacterium triple viable powder, 210 mg per capsule), while the control group will be treated with placebo in the same package. The drug will be taken orally, four capsules at a time, twice a day, and the postoperative cognitive function will be evaluated using a neuropsychological test. If participants request to withdraw from the trial, we will discontinue the allocated intervention for the given trial participants. Adherence to interventions primarily refers to patient self-management adherence. No concomitant care or interventions will be permitted during this trial.

### Participant timeline

Figure 1 presents the schedule for enrollment, interventions, assessments, and visits of participants.

Cognitive function assessment: 1 day before operation: Mini-Mental State Examination (MMSE) screening, digit span test, digit symbol test, Wechsler Memory Scale (WMS), visual regeneration, and digital connection test will be performed. 7 days after operation or before discharge: digit span test, digit symbol test, WMS, visual regeneration, and digital connection tests will be performed again. 7 days after operation: POD will be assessed using the CAM, which includes acute onset, inattention, disorganized thinking, and abnormal consciousness. The diagnosis of delirium by CAM requires the presence of features 1 and 2 and either 3 or 4. Table 2 presents the specific content.

**Table 2:** Specific content of the Confusion Assessment Method

The Confusion Assessment Method (CAM)	
Feature 1	Acute change in mental status with a fluctuating course Is there evidence of an acute change in mental status from the patient’s baseline? Did this behavior fluctuate during the past day, that is, tend to come and go or increase and decrease in severity? This feature is usually obtained from a family member or nurse and is shown by positive responses
Feature 2	Inattention Do the patients have difficulty focusing attention, for example, being easily distractible, or having difficulty keeping track of what was being said?
Feature 3	Disorganized thinking This feature is shown by a positive response to the following question: Was the patient’s thinking disorganized or incoherent, such as rambling or irrelevant conversation, unclear or illogical flow of ideas, or unpredictable switching from subject to subject?
Feature 4	Altered level of consciousness Overall, how would you rate this patient’s level of consciousness? (alert [normal], vigilant [hyperalert], lethargic [drowsy, easily aroused], stupor [difficult to arouse], or coma [unarousable])

The diagnosis of delirium by CAM requires the presence of features 1 and 2 and either 3 or 4

We will also assess the long-term cognitive status using a modified cognitive state telephone interview (TICS-m) 1 month after surgery. TICS-m has been used to screen for dementia and mild cognitive impairment. The TICS-m score ranges from 0 to 48. The higher the score, the better the function. An evaluator will comprehensively evaluate the patient’s condition according to the patient’s medical history, clinical observation, current scale evaluation, and information provided by family members or nursing staff.

### Sample size

According to previous studies, the incidence rate of POCD after a major surgery was 25% [12], and the estimated application of probiotics could reduce it to 10%. According to alpha = 0.05, Power = 0.80, PASS 11 will be applied. The calculated sample size is *n* = 100 cases, with a shedding rate of 10%. The sample size of each group is *n* = 110 cases.

### Data collection and management

This study is a multicenter clinical trial. All data collection physicians will be specially trained by assessors. We will conduct online meetings regularly to share the progress of the trial and discuss the problems that we encounter during the project. Moreover, we will conduct field visits to subcenters for quality control. We shall also organize the trial data regularly to check for any missing data. Furthermore, to promote data quality, we intend to apply other methods and call the participants for follow-up.

Before surgery, the doctor will conduct medical visits for up to 40 min and complete the scale for each patient; furthermore, they will inform the patients that they will be followed up and observed for several consecutive days after surgery. If necessary, the doctor will communicate with the patient through WeChat to understand their latest situation in addition to communicating through phone calls. One day before surgery, the anesthesiologist will record each patient’s general information, including sex, years of education, BMI, dietary habits, history of disease, medication, and surgery. During surgery, blood pressure fluctuations are maintained within 20% of the baseline blood pressure level. Anesthesiologists will record the volume of infusion, estimated blood loss, and urine output and assess the need for a blood transfusion. Similarly, the duration of surgery, anesthesia, and hospital stay after surgery are recorded. After surgery, an analgesia pump will be used for intravenous self-analgesia (uniformly prepared by the pharmacy). Patient-controlled analgesia and or nerve block would be used for postoperative analgesia in all patients to maintain a VAS score of less than 3. The PCIA pump includes the following analgesics: sufentanil citrate 100 μg and flurbiprofen axetil 100 mg which are prepared into 0.9% sodium chloride, in a total volume of 100 ml. All patients receive a 1.5 ml on-demand bolus with a lockout interval of 15 min. The infusion was started immediately after suturing the skin incision and lasted 48 h. We use morphine as rescue drug, which is given according to the patient’s pain situation. At the same time, the drug dosage of the patient’s analgesia pump used and morphine dosage are recorded. Three months after operation, the same doctor will call the patient again for follow-up. All data collected during this clinical study will be entered and/or filed in the patients’ CRFs. The patients’ study participation must be documented appropriately in the CRF with their study number, subject number, date of subject information, informed consent, and date of each visit. Source data will be filed according to the GCP guidelines. The data manager will be responsible for data processing, following the sponsor’s standard operating procedures.

Regular monitoring will be conducted to ensure that the data are adequate, accurate, and complete. The database will be locked only after the completion of quality assurance procedures.

### Statistical methods

#### Data selection for statistical analysis

1. Full analysis set (FAS): According to the principle of intention-to-treat analysis, a full analysis set will include all subjects who will enroll in the study and receive at least one treatment dose.
2. Per-protocol set (PPS): The PPS population will include all FAS patients without major protocol deviations that influence the evaluation of the primary outcome. The efficacy analysis will be performed on FAS and PPS.
3. Safety analysis set (SAS): The safety population consists of all subjects who will receive at least one treatment dose. Safety data analysis will be based on the safety population.

### Statistical analysis

All statistical tests are conducted by two-sided test, and the difference tested will be considered to be statistically significant if *P* ≤ 0.05(unless otherwise specified). The primary outcome measure will be statistically analyzed by chi-square or Fisher exact probability. Secondary outcome measures will be tested by group t-test. The description of quantitative indicators will calculate the mean, standard deviation, median, minimum, and maximum. The number and percentage of patients are used to statistically describe the demographic information, medical history, combined diseases and symptoms, physical examination, operation time, etc. According to the numerical characteristics of variables, *t*-test/Wilcoxon rank sum test is used to compare the quantitative data of age, height, and weight between the two groups; chi-square test/exact probability method is used to compare the categorical variables such as gender, medical history, combined diseases and symptoms, physical examination and so on.

### Additional analyses

The methods used for statistical analyses will also be used for additional analyses of primary and secondary outcomes. Intention-to-treat basis will be used to conduct statistical analysis. The analyses of the outcomes will be analyzed as randomized, regardless of protocol adherence. All variables will be screened for frequency and type of missingness. If missingness is > 5% for any variable, the mean normal value of the patient group will be used for imputation.

No formal interim analysis of primary and secondary outcomes is planned.

### Outcome measures

#### Primary outcome measures

Our primary objective is to assess the incidence of POCD 7 days after surgery. The assessment of cognitive function was completed 1 day before operation and 7 days after operation or on the day of discharge by the following scales: digit span test, digit symbol test, Wechsler Memory Scale (WMS), visual regeneration, and digital connection test. The POCD was defined as follows:

The data of each patient consists of preoperative and postoperative data. The *Z*-score is used to determine whether the patient had POCD [13]. The positive result is at least one *Z*-score ≤ 2 (Wechsler word recall, visual reproduction, number symbols, number width) or ≥ 2 (number lines).

### Secondary outcome measures

Secondary outcomes comprise the following:

1. Incidence of POD

Postoperative delirium will be assessed 7 days after surgery using the Confusion Assessment Method (CAM), which includes acute onset, inattention, disorganized thinking, and abnormal consciousness. The diagnosis of delirium by CAM requires the presence of features 1 and 2 and either 3 or 4 (Table 2).

2) Cognitive status 1 month after surgery

The cognitive status will be determined based on a telephone interview and will be evaluated via TICS-m in 1 month after surgery.

### Oversight and monitoring

#### Composition of the coordinating center and trial steering committee

Diansan Su (DSS), Xiaoxue Hu (XXH), and Jinping Wang (JPW) are present in the trial steering committee. Weifeng Yu (WFY) and Yanhua Zhao (YHZ) are members of the advisory board. Diansan Su (DSS), the principal investigator, is responsible for preparing and revising the protocol and disseminating any changes. Yuwen Chen (YWC) and Xiaoyi Zhang (XYZ) are responsible for coordinating data collection and writing the scientific manuscript. Yizhe Zhang (YZZ) is responsible for supervising the study design and protocol as well as interpretation of the findings. Xiao Zhang (XZ), a statistician, is responsible for performing statistical analyses. Minglan Xu (MLX) and Qi Yao (QY), who are clinical investigators, are responsible for ensuring that the study implementation follows the protocol.

#### Composition of the data monitoring committee, its role, and reporting structure

The need for a data monitoring committee is not considered.

#### Frequency and plans for auditing trial conduct

There are no plans for auditing trial conduct in this investigator-initiated pragmatic trial.

#### Plans for communicating important protocol amendments to relevant parties (e.g., trial participants and ethical committees)

This clinical trial will be conducted according to the ethical committee guidelines. Any problem or protocol modifications during the trial will be communicated to the ethical committee, trial participants, trial registries, journals, and regulators in a timely manner. Ethical committee’s consent will be required to change the protocol.

### Safety evaluation

General safety evaluations will be based on the incidence and type of adverse events (AEs). Safety variables will be tabulated and presented for all patients in the safety set. Adverse events will be coded using World SIVA adverse sedation event reporting tool. The number (%) of subjects with any AEs will be summarized. Probiotics, as beneficial bacteria in the human body, are generally well-tolerated. To date, there has been no evidence that this study may cause any risk or discomfort to participants. However, any harm from the intervention will still be evaluated. Any adverse events, including those voluntarily provided by subjects or obtained through physical examination, laboratory examination, or other examination methods, will be recorded on CRFs and actively handled. We will record any adverse events that occur during the clinical trial, regardless of whether these events were associated with the intervention. Furthermore, all these expected and unexpected trial-related adverse events will be reported in trial publications. A close follow-up will be done until remission or stable condition.

### Provisions for posttrial care

If a participant suffers harm from this trial, he/she will receive free treatment.

### Dissemination plans

The study results will be disseminated via articles published in peer-reviewed journals.

### Plans to provide access to the full protocol, participant-level data, and statistical code

This section is not applicable as we have no plans to provide access to the full protocol, participant-level data, and statistical code.

### Plans for collection, laboratory evaluation, and storage of biological specimens for genetic or molecular analysis in this trial/future use

We have no plans to collect or store biological specimens in this trial.

## Discussion

In recent years, with the aging population, the proportion of elderly patients receiving hip joint and knee joint replacement has increased annually. Due to physiological deterioration, pathological changes, and respiratory, cardiovascular, cerebrovascular diseases, the tolerance of elderly patients to surgery and anesthesia has been greatly reduced. POCD has become a common neurological complication of hip fracture surgery in elderly patients [14]. The reported incidence of POCD varies greatly (9.9–46.1%) [3, 15–20]. Some studies reported that its actual incidence in elderly patients after hip arthroplasty ranges from 16 to 60% [21, 22]. Many studies confirmed that its incidence increases significantly with age, especially in > 65-year-old patients. Its incidence in elderly patients (> 65 years old) is 2–10 times that in young patients. In addition, its incidence in patients aged > 75 years is three times that in patients aged 65–75 years [17]. POCD may last for weeks, months, or years, and may delay recovery, prolong hospital stay, and even increase patient mortality [23, 24].

Intestinal flora is a complex microbial system that exists in human gastrointestinal tract and varies from person to person. It is involved in the regulation of multiple metabolic pathways, signal transduction, and immune inflammatory axis [25]. The microbial characteristics of the human gut at the age of 1 year may predict the cognitive function at the age of 2 years (especially in communication behavior). This may be associated with developmental disorders characterized by cognitive or language delays [26]. Although the body and brain have matured in the middle-aged and elderly, the diversity and stability of the gut microbiota will gradually decrease due to chronic progressive inflammatory reactions, drug use, degradation of digestion and gastrointestinal peristalsis, malnutrition, and decline in immunity during aging. At the same time, aging is accompanied by a decline in brain weight and cognitive function. Aging-related brain morphological changes are common in various aging-related cognitive impairment, such as Alzheimer’s disease [27].

There is increasing evidence that the intestinal microbiota plays a key role in neuropsychiatric diseases and central nervous system function through the gut microbiota-brain axis. In a recent report [28], an abnormal intestinal microbiota composition may be the basis of the mechanism of POCD and POD, indicating that the intestinal microbiota plays a key role in perioperative neurocognitive impairment. The gut-brain axis is a two-way communication system that regulates brain and gut functions [29]. It is well-known that the gut-brain axis consists of the central nervous system, central endocrine system, central immune system, and intestinal microorganisms, including the hypothalamus–pituitary–adrenal axis, sympathetic nervous system, parasympathetic nervous system (vagus nerve), and intestinal nervous system (ENS) of the autonomic nervous system. Signals from the gut can regulate certain brain regions, such as the insula (related to self-cognition), limbic system (related to emotional processing), prefrontal cortex (related to morality), amygdala (related to fear), hippocampus (related to memory), and anterior cingulate gyrus cortex (related to enthusiasm) [30–32]. A normal intestinal mucosal barrier has mechanical, chemical, immune, and biological components. It is composed of intestinal mucosal epithelium, intestinal mucus, intestinal microbiota, secretory immunoglobulin, and gut-associated lymphoid tissue. An abnormal intestinal microbiota is related to the occurrence of the following diseases: irritable bowel syndrome, autism, obsessive–compulsive disorder, and depression. The association between an abnormal intestinal microbiota composition and mental changes caused by these diseases involves the gut-brain axis [33, 34]. The intestinal microbiota changes significantly with age, with increasing proteolytic bacteria and decreasing glycolytic bacteria [35]. Age-related changes in diet, digestion, transport time, colonic pH, and salivary function will affect the intestinal microbiota [36].

Animal studies support the theory that the gut microbiota regulates cognitive symptoms [37], and changes in the gut microbiota are related to social avoidance and depression in mice [38]. Zhan [39] found that anesthesia and surgery induce cognitive dysfunction in male elderly mice, and 24 gut bacteria were significantly altered in mice with POCD compared with those without POCD in which 13 gut bacteria were significantly correlated with escape latency in the Morris water maze test. Yang [40] found that the administration of prebiotics could ameliorate POCD and attenuate surgery-induced neuroinflammation through the gut microbiota-brain axis in rats. Similar trials [11, 41, 42] proved that POCD is significantly associated with an abnormal gut microbiota composition, and abnormalities in specific gut bacteria may be involved in its pathogenesis.

A 2018 systematic review summarized the literature on intestinal microbiota changes associated with cognitive impairment, mild cognitive impairment (MCI), and dementia, evaluated the effects of prebiotics or probiotics on aging animal models and human cognitive symptoms and found that human data were insufficient to make any suggestions [37]. A systematic review of the effects of probiotics on depression published in 2016 showed positive conclusions. Three of these studies found improvements in the MMSE of elderly people taking probiotics [43–45], but all these studies involved participants with previous cognitive dysfunction. Hwang [46] conducted a randomized controlled trial to study the effect of probiotics on the cognition of participants with MCI (*n* = 100). A series of neurocognitive tests were done to assess their cognitive ability 12 weeks after taking a placebo or probiotics. A greater improvement in comprehensive cognitive function was found in the intervention group than in the placebo group (*P* = 0.02). In particular, the intervention group demonstrated a significant improvement in the field of attention. It was also reported [47] that the incidence of POCD in the probiotic group was significantly lower than that in the control group (3 of 59 patients [5.1%] vs. 10 of 61 patients [16.4%], *P* = 0.046).

In our animal experiment, the probiotic regimen of mice refers to the study by Rachmilewitz et al. [48], i.e., from 7 days before surgery to 10 days after surgery. Currently, clinical studies are mainly focused on major abdominal surgery. There is no uniformity in the preoperative administration plan of probiotics, which is given approximately 10 days before surgery or even after surgery [49–51]. These studies have proven that probiotics can effectively reduce postoperative infection rates, fight against intestinal bacterial overgrowth and clinical symptoms, and reduce postoperative complications and hospital stay duration [52]. There are few clinical studies on probiotics for POCD. Wang et al. [47] gave probiotics to patients who underwent noncardiac surgery from the time of admission to discharge and found that probiotics significantly improved postoperative cognitive impairment. In this study, our research participants are elderly patients who will undergo orthopedic elective surgery. As we can only receive official confirmation of a surgical operation 1 day before surgery, we set the preoperative administration route as oral 1 day before surgery. Furthermore, because the average hospital stay duration after surgery is 5–8 days, we set the administration time for patients after surgery as 6 days. Thus, we maximized the duration of oral administration of probiotics for hospitalized patients, which may benefit these patients.

Patients in our study need to receive routine antibiotic treatment, particularly cephalosporins, on the operation day and for 3 days after surgery. To avoid interference with the clinical work of surgeons, we did not limit the use of antibiotics after surgery. Previous studies have reported that probiotic treatment in the presence of antibiotics provides protection against postoperative infectious complications [53–55]. Thus, short-term antibiotic use (several days) is not an obstacle to perioperative probiotic administration. As this is a randomized controlled experiment, the difference in antibiotic treatment would be minimized due to randomization. However, perioperative probiotic treatment may provide improved protection against postoperative cognitive impairment in the absence of antibiotics.

We predict that the perioperative probiotics intervention will alleviate postoperative cognitive impairment in elderly patients undergoing orthopedic surgery. We aim to provide reliable clinical evidence for reducing the rate of postoperative cognitive impairment.

## Trial status

Trial registration: ClinicalTrials.gov, NCT04017403. Registered on Aug. 15, 2019. The protocol version is 1.2 which was approved on Dec. 15, 2019. The recruitment began on Dec.15, 2019, and the approximate date when recruitment will be completed is about Dec. 31, 2023.

## Supplementary Information

**Additional file 1.** SPIRIT Checklist for Trials.

## Data Availability

The participant-level data set cannot be made publicly available because of Chinese data protection rules and regulations. The statistical code is available upon request.

## Abbreviations

POCD: Postoperative cognitive dysfunction
POD: Postoperative delirium
AD: Alzheimer’s disease
GCP: Good clinical practice
CAM: Confusion Assessment Method
TICS-m: Telephone interview for cognitive status—modified
CRF: Case report form
SPIRIT: Standard Protocol Items: Recommendations for Interventional Trials
MMSE: Mini-Mental State Examination
WMS: Wechsler Memory Scale
